# Activated Factor VII–Antithrombin Complex, a Biomarker of Tissue Factor-Related Pathways in Different Clinical Settings: A Narrative Review from Cardiovascular Diseases to Cancer

**DOI:** 10.3390/diagnostics14161711

**Published:** 2024-08-06

**Authors:** Sara Moruzzi, Annalisa Castagna, Marianna Spizzo, Silvia Udali, Patrizia Pattini, Francesca Pizzolo, Simonetta Friso, Nicola Martinelli

**Affiliations:** Department of Medicine, University of Verona, 37134 Verona, Italy; sara.moruzzi@aovr.veneto.it (S.M.); annalisa.castagna@univr.it (A.C.); marianna.spizzo@univr.it (M.S.); silvia.udali@univr.it (S.U.); patrizia.pattini@univr.it (P.P.); francesca.pizzolo@univr.it (F.P.); simonetta.friso@univr.it (S.F.)

**Keywords:** activated factor VII–antithrombin complex (FVIIa–AT), cancer, cardiovascular disease (CAD), coagulation, SARS-CoV-2 infection, preeclampsia, tissue factor (TF), venous thromboembolism (VTE)

## Abstract

Tissue factor (TF) is a transmembrane glycoprotein that represents the fundamental physiological initiator of the coagulation cascade through its interaction with factor VII. TF belongs to the cytokine receptor protein superfamily and contributes to the transduction of cellular signaling. Therefore, TF-related pathways are involved in multiple pathophysiological processes, not only in coagulation/thrombosis but in a wider mechanisms’ panorama, ranging from infective to neoplastic diseases. Consistently, the measurement of TF activity could have a diagnostic and/or prognostic meaning in different clinical conditions. However, the transmembrane localization, the expression on different cellular types and circulating extracellular vesicles, and the different conformations (encrypted and decrypted) and variants (such as the soluble alternatively spliced TF) hamper TF assessment in clinical practice. The activated factor VII-antithrombin (FVIIa–AT) complex is proposed as an indirect biomarker of the TF–FVIIa interaction and, consequently, of the functionally active TF expression. In this narrative review, we evaluate the clinical studies investigating the role of plasma concentration of FVIIa–AT in health and disease. Although without conclusive data, high FVIIa–AT concentrations predict the worst clinical outcomes in different pathologic conditions, such as cardiovascular disease and cancer, thereby suggesting that overactivation of TF-related pathways may play an unfavorable role in various clinical settings.

## 1. The Crucial Roles of Tissue Factor: Haemostasis and Beyond

**Figure 1:**
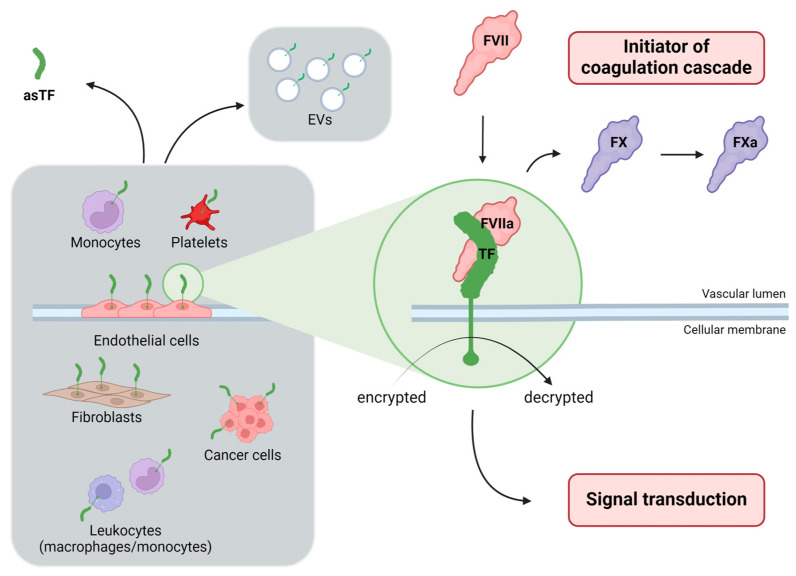
The multifaced expression and role of tissue factor (TF). In the context of the crossroad between coagulation cascade and signal transduction, the various cellular sources of TF (on the left), the different isoforms—encrypted and decrypted—(in the middle), and the alternatively spliced forms (soluble and asTF) are shown. Created with BioRender.com. asTF, alternatively spliced tissue factor; EVs, extracellular vesicles; TF, tissue factor.

Tissue factor (TF) is a transmembrane glycoprotein belonging to the cytokine receptor protein superfamily, historically known as the initiator of the extrinsic pathway of the coagulation cascade and the major cellular activator of the clotting cascade [1]. The crucial role of TF is well illustrated by the reports that, by attempting to generate TF knock-out mice models, have shown a severe lethality of TF−/− embryos, thus suggesting that TF is biologically indispensable for life [1]. The full-length, transmembrane TF molecule is formed by three different domains: (i) an extracellular domain at the N-terminal involved in the interaction with coagulation factor VII (FVII), (ii) a transmembrane domain anchoring TF to the membrane, and (iii) a cytoplasmatic C-terminal domain involved in signal transduction [2]. The full-length TF is typically expressed on vascular smooth muscle cells, fibroblasts, and perivascular cells, where it forms a haemostatic barrier that leads toward a rapid initiation of the coagulation cascade when the blood vessel is damaged [3]. Moreover, TF is expressed by activated leukocytes (monocytes/macrophages) and by stimulated endothelial cells [4], although there are some controversies on the expression of TF by endothelial cells in vivo [5]. Full-length TF can also be found in blood on extracellular vesicles (EVs) derived from a variety of cell types, including platelets, monocytes, and endothelial cells [6], as well as from malignant tumor cells [7] which are further important sources expressing TF [8]. The cellular sources of TF expression are, therefore, multiple and heterogeneous (for a review see Grover SP & Mackman N, Arterioscler Thromb Vasc Biol. 2018 [9]), as summarized in Figure 1.

**Figure 2:**
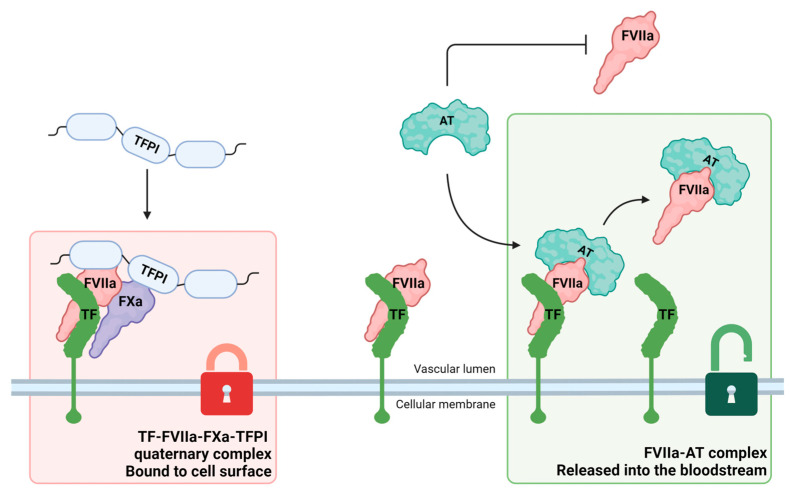
Modes of action of the main inhibitors of the tissue factor (TF) pathway. Tissue factor pathway inhibitor (TFPI) forms a tetramolecular complex (TF–FVIIa–FXa–TFPI) which remains stable on the surface of the cell membrane (on the left). Antithrombin (AT) reacts with FVIIa only if FVIIa is bound to functionally active TF, thereby forming a FVIIa–AT complex, which is released from the cell membrane into the bloodstream (on the right). Created with BioRender.com. AT, antithrombin; FVIIa, activated factor VII; FXa, activated factor X; FVIIa–AT, activated factor VII-antithrombin; TF, tissue factor; TFPI, tissue factor pathway inhibitor.

TF is usually expressed on the cellular surface in a quiescent or “encrypted” form, while activation or “decryption” is necessary for procoagulant activity [10]. Experimental observations suggested that decryption involves both the formation of a disulfide bond between unpaired cysteine residues 186 and 209 in encrypted TF and the exposure of phosphatidyl serine on the cellular surface [11]. When functionally active TF is exposed to the bloodstream, it encounters either FVII, converting it to its activated form (FVIIa), or binds FVIIa [12]. Once the TF–FVIIa complex is formed, it can initiate the blood clotting cascade by activating factor X (FX). However, the role of TF is notably not limited to haemostatic function and contributes to signaling in a variety of cells, modulating inflammatory pathways and cellular proliferation [9]. Two different plasmatic inhibitors can interact with the TF–FVIIa complex: tissue factor pathway inhibitor (TFPI) and antithrombin (AT) [12]. The main inhibitor of the TF pathway is TFPI, which first binds to activated FX (FXa) and subsequently forms a tetramolecular complex (TF–FVIIa–TFPI–FXa), which remains stable on the surface of the cell membrane. Moreover, AT can interact with TF–FVIIa forming a FVIIa–AT complex, which detaches from TF and is released from the cell membrane into the bloodstream [12] (Figure 2).

Post-transcriptional regulations of TF have also been studied. It is worth noting that an alternatively spliced TF (asTF), which lacks the transmembrane domain and presents an altered C-terminus domain due to the deletion of exon 5, has been described [13]. Alternatively spliced TF is a soluble molecule that can be detected in the bloodstream, although its role remains partially undefined. Remarkably, asTF lacks residues required for interaction with FX and therefore has a reduced procoagulant activity. There is evidence suggesting, however, that asTF may have a greater role in angiogenesis and cancer biology rather than in coagulation and thrombosis [14], thereby favouring tumor growth and metastasis spreading [15,16].

At a post-transcriptional level, several microRNAs (miRNAs) have been reported to modulate TF synthesis in physiological but mostly pathological conditions. Interestingly, miRNAs targeting TF have been then proposed as biomarkers of TF pathway with even potential therapeutic application [17,18].

## 2. The Difficulty of Evaluating Tissue Factor Activity: Biomarkers Searching for an Elusive Molecule

The multiform and differentiated TF expression explains the difficulties of the laboratory assessment of its activity. According to its transmembrane cellular localization, the direct determination of TF in plasma is not and cannot be a routine assessment in clinical practice. Notably, blood-born TF, if present, is at very low concentration and without procoagulant functional activity [9]. The soluble asTF can be detected in plasma but has negligible procoagulant activity [19]. On the other hand, the full-length TF can be isolated and assessed from EVs released into the bloodstream [20]. Importantly, antigen-based assays cannot differentiate between the two conformational forms of the full-length TF, encrypted (with low procoagulant activity) or decrypted (with high procoagulant activity), and therefore is not an accurate way to measure TF activity [20].

## 3. Activated Factor VII-Antithrombin Complex as an Indirect Biomarker of Tissue Factor Pathway

The plasma levels of FVIIa–AT have been proposed to indirectly reflect TF–FVIIa interaction and thus TF expression/activity. The biological basis of this assumption refers to biochemical studies that documented the molecular mechanisms by which AT can inhibit the TF–FVIIa complex. The AT reaction with FVIIa was first reported in 1974 [21]. Later, in 1993, it was shown that the interaction between TF–FVIIa greatly enhances the susceptibility of FVIIa to inhibition by AT, thereby suggesting that AT reacts with FVIIa only if FVIIa is bound to functionally active TF [22]. In 1995 it was shown that the decrease in FVIIa levels associated with TF in the presence of AT was the result of the accelerated dissociation of FVIIa from TF after the binding of AT to the TF–FVIIa complexes, thereby suggesting that FVIIa–AT complexes released into the blood correlate directly with functionally active TF expressed [23]. Notably, AT inhibition of TF–FVIIa interaction does not require the presence of FXa, while TFPI cannot inhibit TF–FVIIa interaction without the presence of FXa.

Consistent with these biochemical premises, the FVIIa–AT complex could be considered as an indirect biomarker of the overall TF expression and was seen as a hypothetically useful tool for the assessment of TF activity in health and disease [12]. In 2003, the first patent of an enzyme-linked immunosorbent assay (ELISA) for measuring FVIIa–AT plasma levels was published [24]. Subsequently, some companies developed and commercialized specific ELISA assays with this aim, thus paving the way for more extensive population-based studies in order to assess the clinical significance of the measurement of FVIIa–AT plasma levels.

## 4. Plasma Concentration of Activated Factor VII-Antithrombin Complex in Different Clinical Settings in Humans

**Figure 3:**
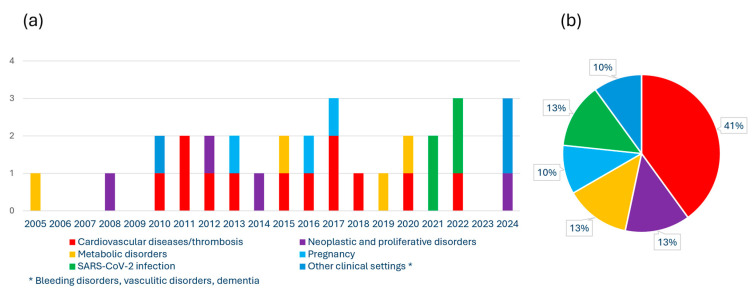
Studies investigating plasma-activated factor VII–antithrombin complex (FVIIa–AT) in humans. (**a**) Distribution of the literature across timeline (20 years); (**b**) representation of prevalence (%) of the studies according to the different clinical settings.

Thirty studies during the last two decades (Figure 3a) have assessed FVIIa–AT plasma concentration in humans, investigating the possible diagnostic and/or prognostic role of this biomarker in different clinical settings.

The majority of these studies focused on cardiovascular diseases/thrombosis and neoplastic disorders (Figure 3b). A detailed description of the main results of these research papers is presented in the following paragraphs and summarized in Table 1.

### 4.1. Thrombosis and Cardiovascular Disease

The first paper highlighting the indeed plausible association between FVIIa–AT plasma concentrations and thrombotic diseases was published in 2010 by Spiezia and colleagues who found that FVIIa–AT complex levels were lower in patients with acute thrombosis, either arterial or venous, than in those with previous thrombosis or in healthy controls [25]. FVIIa–AT levels correlated strongly and positively with plasma FVIIa activity, which is considered more specific and reliable than FVII coagulant activity [26]. On the other hand, no relevant role of AT plasma levels was observed in predicting FVIIa–AT variability [25]. In a subsequent study conducted on patients with acute deep vein thrombosis (DVT) by using an in-house assay, the FVIIa–AT complex was detected only in a minority of patients (about 10%) and controls (about 30%). In this small subgroup of subjects with detectable FVIIa–AT, the related levels were substantially higher in patients with DVT than in controls [27].

In the setting of portal vein thrombosis (PVT), with or without concomitant liver cirrhosis, it was shown that non-cirrhotic patients with PVT had higher FVIIa–AT levels than healthy controls. On the other hand, no significant differences in FVIIa–AT levels were found between cirrhotic patients with or without PVT, with both groups characterized by lower levels of FVIIa–AT as compared with non-cirrhotic subjects [28]. Noteworthily, in the group of cirrhotic patients characterized by low synthesis of plasma proteins, including FVII and AT, there was a significant positive correlation of FVIIa–AT with both FVII and AT [28]. Similarly, another study investigating HCV-related cirrhosis with or without PVT showed no significant difference in FVIIa–AT levels between cirrhotic patients with or without PVT. Cirrhotic patients with PVT had significantly lower FVIIa–AT levels compared to either non-cirrhotic patients with PVT or healthy controls [29]. These results indicated mainly the presence of low levels of FVIIa–AT in patients with acute thrombotic complications, as well as in those with liver cirrhosis. There was also a strong correlation between FVIIa–AT and FVII/FVIIa levels. Therefore, it appears biologically plausible that conditions which are associated with low FVII/FVIIa levels because of either reduced synthesis (like liver cirrhosis) or increased consumption (like acute thrombosis) can affect FVIIa–AT plasma concentration. Moreover, it is tempting to speculate that such conditions may represent a major confounding factor in the interpretation of FVIIa–AT plasma levels as an indirect biomarker of TF expression and activity.

In 2012, the investigations on the potential clinical significance and prognostic role of FVIIa–AT were extended to cardiovascular disease (CVD). The Stockholm Coronary Atherosclerosis Risk Factor (SCARF) study analysed 200 patients with ischemic heart disease and previous (3–6 months before) myocardial infarction (MI). FVIIa–AT levels were slightly higher in patients with previous MI than in controls even though with a substantial overlap of values [30]. Furthermore, in the Stockholm prospective study of CVD, FVIIa–AT levels had no predictive value for the development of future CVD during a 5-to-7-year follow-up in 60-year-old individuals [30]. Afterwards, in the Italian observational study cohort of the Verona Heart Study (VHS), investigating subjects with or without angiographically demonstrated coronary artery disease (CAD), no difference in FVIIa–AT levels between CAD and CAD-free subjects was found. However, within the group of 546 patients with clinically stable CAD (acute coronary syndrome, as well as any recent acute illness, were exclusion criteria for VHS), during a 64-month median follow-up, subjects with high FVIIa–AT levels (higher than the median value at baseline) had a two-fold greater risk of both total and cardiovascular mortality, independent of traditional confounding factors [31]. Moreover, high FVIIa–AT levels were associated with increased thrombin generation, thereby indicating FVIIa–AT as a marker of prothrombotic diathesis [31]. In a subgroup analysis within the same population, consistent with its role as a thrombophilic biomarker, high FVIIa–AT plasma levels also correlated directly with FXa generation and, notably, the characteristics of hypercoagulability related to FVIIa–AT were specifically detectable in the initiation phase of coagulation [32]. The potential role of FVIIa–AT as a prognostic biomarker was confirmed in the larger cohort of the Cardiovascular Health Study (CHS), a prospective cohort study investigating risk factors for coronary heart disease and stroke [33]. Within the CHS cohort, high levels of FVIIa–AT were associated with an increased risk of mortality from all causes in adults aged 65 years and older. The almost 3500 subjects of the CHS also allowed for a genome-wide association study (GWAS) on genetic determinants of FVIIa and FVIIa–AT in European Americans. The most significant SNP for FVIIa–AT (and also FVIIa) was rs1755685 in the F7 promoter region on chromosome 13, while there was also an association with rs867186 located in PROCR on chromosome 20 [33].

In a further smaller cohort study on 120 CAD patients, FVIIa–AT levels were positively associated with current smoking and multivessel CAD. More relevant from a clinical point of view, CAD patients with a high baseline FVIIa–AT plasma concentration had an increased risk of both ischemic stroke/systemic thromboembolism and the composite endpoint of myocardial infarction, stroke/systemic thromboembolism, and cardiovascular death during a median follow-up of 106 months [34]. FVIIa–AT levels have also been assessed in the setting of cardiac surgery. In a small study of 13 adult patients undergoing cardiac surgery with cardiopulmonary bypass, the pre-surgery concentrations of FVIIa–AT rose about twice as much post-heparin bolus and those higher concentrations were maintained throughout the cardiopulmonary bypass and post-protamine administration time so that it was suggested that AT appeared to play an active role in FVIIa inhibition during cardiac surgery [35]. This small study was not able to distinguish between an increase in TF exposure due to surgery and an increase in the AT binding to FVIIa mediated by heparin, although the latter hypothesis seems suggestive. On the other hand, no definite conclusions could be drawn on a possible prognostic significance of FVIIa–AT levels in this clinical setting. As it refers to cerebrovascular disease, in a study on paediatric patients including 54 children with ischemic stroke (IS) and 7 children with cerebral vein thrombosis (CVT), FVIIa–AT levels were lower in children with acute IS (n = 38) than in healthy children, while those with previous IS (n = 16) or CVT had levels of FVIIa–AT not significantly higher than healthy children [36]. In this paediatric population, FVIIa activity plasma levels were directly correlated with FVIIa–AT plasma levels [36]. FVIIa–AT levels were significantly lower also in 33 adult subjects with acute ischemic stroke (of whom 13 underwent thrombolysis with rtPA) than in age-matched controls [37].

### 4.2. Neoplastic and Proliferative Disorders

A study assessing 93 patients with haematological malignancies (acute myeloid leukaemia, chronic lymphatic leukaemia, multiple myeloma, non-Hodgkin’s lymphoma) found no difference in baseline FVIIa–AT plasma concentrations between haematological cases and healthy controls. On the other hand, FVIIa–AT levels increased during the follow-up after cancer treatment [38]. In the few haematological patients (n = 4) who had a previous venous thrombosis (VT), FVIIa–AT levels were lower at baseline, while those with VT after diagnosis of malignancy (n = 8) had no difference in FVIIa–AT levels as compared with patients without malignancy [38]. In contrast, a study on essential thrombocythemia, a well-known chronic myeloproliferative neoplasm, showed different results [39]. FVIIa–AT levels were significantly higher in 73 patients with essential thrombocythemia, in particular in those positive for the JAK2V617F mutation (n = 37), as compared with controls [39]. With regard to solid cancers, a study including 60 patients with different types of active cancer (gastro-intestinal, breast, pancreatic, prostatic, lung, liver, cerebral) with or without acute venous thromboembolism (VTE) showed that patients with active cancer and without acute VTE had significantly higher FVIIa–AT levels than controls, while patients with acute VTE, either unprovoked or with active cancer, had lower FVIIa–AT levels than controls [40].

In a recent study on 136 Italian patients with different types of liver cancer, i.e., hepatocellular carcinoma, cholangiocarcinoma, and metastasis of colon cancer, FVIIa–AT plasma concentrations were more elevated in cancer patients than among sex- and age-matched controls and differed among the specific types of cancer with the highest levels in patients with cholangiocarcinoma [41]. Most importantly, high FVIIa–AT levels were associated with high TF mRNA levels in cancer tissue, thus supporting the concept of FVIIa–AT as a reliable marker of TF expression at the cellular level [41]. Finally, high FVIIa–AT levels predicted an increased risk of mortality in the cancer population during a 34-month median follow-up, thereby suggesting that FVIIa–AT may allow the identification of patients with cancer characterized by an enhanced TF expression and a greater risk of mortality [41].

### 4.3. Metabolic Disorders

A series of studies assessed FVIIa–AT levels also in the setting of metabolic disorders, which are well recognized as traditional cardiovascular risk factors. A study evaluating both FVIIa–AT and thrombin generation was performed in 80 overweight and obese patients, asymptomatic for thrombotic events, i.e., 20 overweight subjects (BMI range 25–29.9 kg/m^2^), 20 subjects with I degree obesity (BMI range 30–34.9 kg/m^2^), 20 subjects with II degree obesity (BMI range 35–39.9 kg/m^2^), and 20 subjects with III degree obesity (BMI ≥ 40 kg/m^2^). No significant difference in FVIIa–AT levels was found between overweight/obese patients and controls [42]. On the other hand, the obese patients showed higher peak thrombin as compared to controls [42]. In a small controlled cross-over study on 20 obese patients, in whom high-fat and low-fat meals were served, FVIIa–AT levels, along with FVIIa and triglycerides, increased significantly after high-fat meals and low-fat meals with the largest increase after high-fat meals [43].

Within the frame of the Italian angiographically-controlled study cohort of the Verona Heart Study (VHS), the plasma concentration of FVIIa–AT significantly and directly correlated with total and high-density lipoprotein cholesterol, triglycerides, ApoA-I, ApoC-III, and ApoE levels [44]. More precisely, ApoC-III showed the strongest correlation with FVIIa–AT levels, even in multiple adjusted models [44]. FVIIa–AT complex was also used as a tool to explore TF expression in different experimental settings. In a study including nine patients with type 1 diabetes who received 20 infusions of isolated human islets of Langerhans, FVIIa–AT levels increased rapidly within 15–60 min after islet infusion and correlated negatively with the change in C-peptide over 7 days [45]. Based on these data and further results, it was hypothesized that TF produced by the cells of the islets of Langerhans elicited an instant blood-mediated inflammatory reaction, thereby being associated with a negative outcome of clinical islet transplantation [45].

### 4.4. SARS-CoV-2 Infection

In the last years, the pandemic caused by SARS-CoV-2 infection renewed interest in a better understanding of the complex interconnections between immune response, inflammation, clotting, and thrombosis, which are now collected under the term immunothrombosis. Acute and severe infections, like those leading to septic shock, are well known to be generally associated with several alterations in coagulation mechanisms, including an increase in the expression and activity of TF [46,47]. However, to the best of our knowledge, the specific assessment of FVIIa–AT during infectious diseases was performed just in patients with SARS-CoV-2 infection so far. Francischetti and colleagues analysed 66 adult COVID-19 patients at the Johns Hopkins Hospital having moderate (n = 40) and severe disease (n = 26). FVIIa–AT levels were significantly higher in COVID-19 patients with severe disease than in controls negative for SARS-CoV-2 infection, with intermediate levels in COVID-19 patients with moderate disease [48]. Similar results were shown in an Italian case-control study on 40 subjects with SARS-CoV-2 pneumonia during the first pandemic wave, compared to 40 historic non-COVID-19 controls with inflammation and 40 historic non-COVID-19 controls without inflammation [49]. FVIIa–AT levels were higher in COVID-19 patients than in non-COVID-19 subjects, either with or without inflammation, while no difference was observed among non-COVID-19 subjects [49]. In 203 Dutch patients with previous acute COVID-19, FVIIa–AT levels were still increased in a third of the patients (35%) 6–20 weeks after recovery, confirming a sustained and long-term activation of coagulation mechanisms in COVID-19 [50]. FVIIa–AT plasma concentration was also measured, by means of an in-house developed assay, in 40 adult healthcare workers before and after (1–2 days) the first dose of ChAdOx1 vaccination against SARS-CoV-2 [51]. FVIIa–AT levels were significantly decreased after vaccination suggesting that, generally, the first dose of ChAdOx1 vaccination does not lead to an activation of the TF pathway [51].

**Table 1:** Studies assessing plasma levels of FVIIa–AT in humans in different clinical settings.

**Thrombosis and Cardiovascular Disorders (CVD)**			
**Type of Study**	**Study Population**	**FVIIa–AT-Related Main Results**	**Reference**
Case-control study	- 154 patients with either arterial or venous thrombosis (48 with acute thrombosis, 16 arterial and 32 venous; 106 with previous thrombotic events, 45 arterial and 61 venous) - 154 healthy controls - 160 subjects with specific clinical condition (53 with inherited FVII deficiency; 58 with antithrombin (AT) deficiency; 49 taking VKAs with PT-INR 2–3)	(i) FVIIa–AT levels were lower in patients with either acute arterial or venous thrombosis than either subjects with previous thrombosis or healthy controls. The difference was statistically significant only between acute and previous thrombosis. (ii) FVII levels as crucial determinant of FVIIa–AT levels (strong positive correlation between FVII-AT and FVII/FVIIa levels), while the role of AT was no significant.	Spiezia L et al., 2010 [25]
Case-control study	- 54 children with ischemic stroke (IS), 38 during the acute phase within 72 h and 16 with a previous IS at least 3 months before blood sample collection - 7 children with previous cerebral vein thrombosis (CVT) - 24 healthy children	(i) FVIIa–AT levels were lower in children with acute IS than in healthy children (ii) Children with previous IS or CVT had no significantly higher FVIIa–AT levels than healthy children. (iii) FVIIa levels were directly correlated with FVIIa–AT levels.	Spiezia L et al., 2011 [36]
Cohort study	- 13 adult patients undergoing cardiac surgery with cardiopulmonary bypass	(i) The presurgery concentrations of FVIIa–AT rose about twofold postheparin bolus and then was maintained throughout cardiopulmonary bypass and post-protamine administration. (ii) AT appears to play an active role in FVIIa inhibition during cardiac surgery.	Davidson SJ and Woodhams B, 2011 [35]
Case-control study and Cohort Study with nested case-control design	*Stockholm Coronary Atherosclerosis Risk Factor (SCARF) study* - 200 patients with ischemic heart disease post myocardial infarction (MI) and 340 controls *Stockholm prospective study of 60-year-olds individuals* - 211 incident CVD cases and 633 controls	(i) FVIIa–AT levels were slightly increased after MI but with a substantial overlap of values. (ii) FVIIa–AT levels had no predictive value for the development of future CVD.	Silveira A et al., 2012 [30]
Case-control study	- 45 patients with portal vein thrombosis (PVT), of whom 33 cirrhotic PVT and 12 noncirrhotic PVT, - 45 sex- and age-matched controls, of whom 33 with liver cirrhosis and 12 healthy volunteers	(i) FVIIa–AT levels were significantly higher in noncirrhotic PVT patients than in controls. (ii) No significant difference in FVIIa–AT levels was seen between cirrhotic patients with or without PVT. (iii) Cirrhotic patients with or without PVT had a significant reduction of FVIIa–AT levels than in noncirrhotic subjects. (iv) Both FVII and AT levels were directly correlated with FVIIa–AT levels.	Rossetto V et al., 2013 [28]
Case-control study	- 148 patients with acute deep venous thrombosis (DVT) - 179 controls	FVIIa–AT plasma concentration was measured using an in-house developed assay and was detected only in a minority of patients (n = 16, 11%) and controls (n = 58, 33%). In these individuals the related levels were substantially higher in patients with DVT than in controls.	Schut AM et al., 2015 [27]
Case-control study and Longitudinal Cohort study	*Verona Heart Study (VHS)* - 686 subjects with or without angiographically demonstrated coronary artery disease (CAD): 546 subjects with clinically stable CAD—subjects with acute coronary syndrome were excluded—and 140 CAD-free subjects	(i) FVIIa–AT levels correlated directly with peak and endogenous thrombin potential in thrombin generation analysis. (ii) No difference in FVIIa–AT levels between CAD and CAD-free subjects. (iii) Within the CAD population, during a 64-month median follow-up, patients with high FVIIa–AT levels (higher than the median value at baseline) had a two-fold greater risk of both total and cardiovascular mortality, independent of confounding factors.	Martinelli N et al., 2016 [31]
Case-control study	- 33 subjects with acute IS, of whom 13 underwent thrombolysis with rtPA and 20 did not go to thrombolysis; - 20 age-matched controls	FVIIa–AT levels were significantly lower in patients with IS than in controls.	Slomka A et al., 2017 [37]
Case-control study	- 30 HCV-cirrhosis patients with PVT - 35 HCV-cirrhotic patients without PVT - 15 non-cirrhotic patients with PVT - 15 healthy volunteers	(i) No significant difference in FVIIa–AT levels between cirrhotic patients with or without PVT. (ii) Cirrhotic patients had significantly lower FVIIa–AT levels compared to non-cirrhotic patients with PVT and healthy controls.	Abu El-Makarem MA et al., 2017 [29]
Prospective Cohort studyGenome-wide association study (GWAS) of FVIIa and FVIIa–AT	*Cardiovascular Health Study (CHS)* in European-Americans adults aged 65-years and older - 3427 subjects with available data of FVIIa–AT	(i) The most significant SNP for FVIIa–AT (and also FVIIa) was rs1755685 in the F7 promoter region on chromosome 13; there was also an association with rs867186 located in PROCR on chromosome 20. (ii) High levels of FVIIa–AT were associated with an increased risk of mortality from all causes.	Olson NC et al., 2018 [33]
Observational study	- 40 male patients with clinically stable CAD	(i) FVIIa–AT levels correlated directly with FXa generation. (ii) FVIIa–AT-related features of hypercoagulability were specifically detectable in the initiation phase of coagulation.	Baroni M et al., 2020 [32]
Longitudinal Cohort study	- 120 patients with CAD	(i) FVIIa–AT levels were positively associated with current smoking and multivessel CAD. (ii) During a median follow-up of 106 months, CAD patients with high baseline FVIIa–AT levels had an increased risk of IS/systemic thromboembolism and a composite endpoint of MI, stroke/systemic thromboembolism, and cardiovascular death.	Paszek E et al., 2022 [34]
**Neoplastic and Proliferative Disorders**			
**Type of Study**	**Study Population**	**FVIIa–AT-Related Main Results**	**Reference**
Case-control study	- 93 patients with haematological malignancies (acute myeloid leukaemia, chronic lymphatic leukaemia, multiple myeloma, non-Hodgkin’s lymphoma), of whom 4 who had symptomatic venous thrombosis (VT), 8 with subsequent VT after diagnosis of malignancy - 69 healthy controls	(i) There was no difference in baseline FVIIa–AT plasma levels between cases and controls. (ii) FVIIa–AT levels increased after cancer treatment. (iii) Four patients who had a symptomatic VT had lower levels of FVIIa–AT at baseline, while eight patients with subsequent VT after diagnosis of malignancy had no difference in levels of FVIIa–AT as compared with patients without VT.	Negaard HFS et al., 2008 [38]
Retrospective case-control study	- 30 patients with active cancer (10 gastro-intestinal, 6 breast, 5 pancreatic, 3 prostatic, 3 lung, 2 liver, 1 cerebral) without acute venous thromboembolism (VTE) - 30 patients with active cancer (8 gastro-intestinal, 6 lung, 5 pancreatic, 5 prostatic, 3 breast, 2 uterine, 1 cerebral) and acute VTE - 30 patients with unprovoked VTE and 90 controls	(i) Patients with active cancer and without acute VTE had significantly higher FVIIa–AT levels than controls. (ii) Patients with acute VTE either unprovoked or with active cancer had lower FVIIa–AT levels than controls.	Spiezia L et al., 2012 [40]
Case-control study	- 73 patients with essential thrombocythemia, of whom 37 positive for the JAK2V617F mutation - 72 controls	FVIIa–AT levels were significantly elevated in patients with essential thrombocythemia, in particular those positive for the JAK2V617F mutation, as compared with controls.	Marchetti M et al., 2014 [39]
Case-control study and Longitudinal Cohort study	- 136 cancer patients (52 hepatocellular carcinoma, 41 cholangiocarcinoma, and 43 colon cancer) - 136 sex- and age-matched cancer-free subjects	(i) FVIIa–AT levels were higher in cancer patients than in controls. (ii) FVIIa–AT levels were different among the specific types of cancer. (iii) High FVIIa–AT levels were associated with high TF mRNA levels in cancer tissues. (iv) High FVIIa–AT levels were associated with an increased risk of mortality after a 34-month median follow-up.	Martinelli N et al., 2024 [41]
**Metabolic Disorders**			
**Type of Study**	**Study Population**	**FVIIa–AT-Related Main Results**	**Reference**
Experimental study	- 9 patients with type 1 diabetes received 20 infusions of isolated human islets of Langerhans	(i) TF produced by the cells of the islets of Langerhans elicited instant blood-mediated inflammatory reaction and was associated with a negative outcome of clinical islet transplantation. (ii) FVIIa–AT increased rapidly within 15–60 min after islet infusion and FVIIa–AT levels correlated negatively with the change in C-peptide over 7 days.	Johansson H et al., 2005 [45]
Case-control study	- 80 overweight and obese subjects asymptomatic for thrombotic events (20 overweight subjects, 20 subjects with I degree obesity, 20 subjects with II-degree obesity, and 20 subjects with III degree obesity)–40 normal weight controls	(i) No significance increase in FVIIa–AT complex in overweight and obese patients compared to controls. (ii) No significant increase in FVIIa–AT levels in any patient group compared to controls. (iii) Obese patients had higher peak thrombin compared to controls.	Campello E et al., 2015 [42]
Observational study	*Verona Heart Study (VHS)* - 666 subjects with (535) or without (131) angiographically-demonstrated CAD and complete laboratory data of plasma lipids and apolipoprotein profile	(i) Plasma concentration of FVIIa–AT significantly and directly correlated with total and high-density lipoprotein cholesterol, triglycerides, ApoA-I, ApoC-III and ApoE levels. (ii) ApoC-III showed the strongest correlation with FVIIa–AT levels, even in multiple adjusted models.	Martinelli N et al., 2019 [44]
Controlled cross-over study	20 obese patients, in whom high-fat and low-fat meals were served	(i) FVIIa–AT levels (along with FVIIa and triglycerides) increased significantly after high-fat meals and low-fat meals with the largest increase after high-fat meals.	Landgrebe LE et al., 2020 [43]
**SARS-CoV-2 Infection**			
**Type of Study**	**Study Population**	**FVIIa–AT-Related Main Results**	**Reference**
Case-control study	- 66 adult COVID-19 patients (40 with moderate disease and 26 with severe disease) - 9 controls negative for SARS-CoV-2 infection	FVIIa–AT levels were significantly higher in COVID-19 patients with severe disease than in controls, with intermediate levels in COVID-19 patients with moderate disease.	Francischetti IMB et al., 2021 [48]
Cohort study	- 40 adult health care workers, in whom blood samples were drawn before and after (1–2 days) the first dose of ChAdOx1 vaccination against SARS-CoV-2	FVIIa–AT plasma levels (measured using an in-house developed assay) were significantly decreased after vaccination.	Willems LH et al., 2021 [51]
Cross-sectional Cohort study	- 203 patients with prior COVID-19 (6–20 weeks after recovery of acute COVID-19) and 312 historic controls	FVIIa–AT levels were increased in a third of the patients (35%) 6–20 weeks after recovery of acute COVID-19.	Willems LH et al., 2022 [50]
Case-control study	- 40 subjects with SARS-CoV-2 pneumonia during the first pandemic wave - historic non-COVID-19 controls: 40 with inflammation and 40 without inflammation	FVIIa–AT levels were higher in COVID-19 patients than in non-COVID-19 subjects, either with or without inflammation, while no difference was observed among non-COVID-19 subjects.	Martinelli N et al., 2022 [49]
**Pregnancy and Preeclampsia**			
**Type of Study**	**Study Population**	**FVIIa–AT-Related Main Results**	**Reference**
Case-control study	- 105 pregnant women (30 in the first trimester, 30 in the second trimester, 30 in the third trimester and 15 with pre-eclampsia) - 105 healthy women	(i) FVIIa–AT levels were significantly higher in pregnant than in healthy women. (ii) no significant difference in FVIIa–AT levels between pregnant women in the third trimester and those with pre-eclampsia. (iii) women with pre-eclampsia had significantly higher FVIIa–AT/FVIIa ratio than pregnant women in the third trimester.	Spiezia L. et al., 2013 [52]
Case-control study	- 108 severe preeclamptic women - 104 normotensive pregnant women	FVIIa–AT levels were not different between severe preeclamptic and normotensive pregnant women.	Alpoim PN et al., 2016 [53]
Cohort study	- 26 pregnant with severe early (gestational age < 34 weeks) preeclampsia - 19 pregnant with severe late (gestational age ≥ 34 weeks) preeclampsia	Increased FVIIa–AT levels were found in early severe preeclampsia compared to late severe preeclampsia.	Dusse LMS et al., 2017 [54]
**Other Clinical Settings**			
**Type of Study**	**Study Population**	**FVIIa–AT-Related Main Results**	**Reference**
Cohort study for a population pharmacokinetic model	- 10 subjects with severe FVIII or FIX deficiency treated with recombinant human factor VIIa (rFVIIa)	The rFVII–AT complex formation was responsible for 65% of the total rFVIIa clearance. The formation of rFVIIa–AT complex was able to explain the difference observed between the rFVIIa:C and the rFVII:Ag concentration.	Agerso H et al., 2010 [55]
Longitudinal Cohort study	- 71 patients with antineutrophil cytoplasmic antibody (ANCA)-associated vasculitis, 4 of them (6%) had history of VTE and 5 had VTE symptomatic after presentation. - Coagulation parameters assessed at presentation with active disease and at follow-up after 6 months of treatment	(i) FVIIa–AT levels were normal in the majority of patients (90%) at baseline and further slightly decreased over time. (ii) No difference in FVIIa–AT levels between patients with or without VTE.	Busch MH et al., 2024 [56]
Prospective Cohort study	*Cardiovascular Health Study (CHS)* in adults—3185 older adults (aged 65-years and older) free of dementia in 1992/1993	There was no evidence of linear associations between FVIIa–AT levels with any dementia risk.	Harrington LB et al., 2024 [57]

Abbreviations: CVD, Cardiovascular Disorders; AT, Antithrombin; IS, Ischemic Stroke; CVT, cerebral vein thrombosis; MI, myocardial infarction; PVT, portal vein thrombosis; DVT, deep venous thrombosis; CAD, coronary artery disease; VT, venous thrombosis; VTE, acute venous thromboembolism; rFVIIa, recombinant human factor VIIa.

### 4.5. Pregnancy and Preeclampsia

The modifications of coagulation during pregnancy, usually involving a shift toward a hypercoagulable state, are well known, as well as the potential role of blood clotting in many pregnancy-related diseases. The first study investigating FVIIa–AT levels in pregnancy analysed 105 pregnant women, 30 in the first trimester, 30 in the second trimester, 30 in the third trimester, and 15 with pre-eclampsia [52]. Higher FVIIa–AT plasma levels were found in pregnant women than in healthy non-pregnant women. Women with pre-eclampsia had a significantly higher FVIIa–AT/FVIIa ratio than pregnant women in the third trimester, but no significant difference in FVIIa–AT levels between pregnant women in the third trimester and those with pre-eclampsia was found [52]. Similarly, in a cohort study comparing the concentration of FVIIa–AT between 108 severe preeclamptic women and 104 normotensive pregnant women, FVIIa–AT levels were not different between severe preeclamptic and normotensive pregnant women [53]. Finally, increased FVIIa–AT levels have been detected in 26 pregnant women with early (gestational age < 34 weeks) severe preeclampsia compared to 19 with late (gestational age ≥ 34 weeks) severe preeclampsia, thus suggesting a higher TF-related hypercoagulability in early preeclampsia [54].

### 4.6. Other Clinical Settings

The assessment of FVIIa–AT plasma levels has also been used in other clinical contexts and diseases. In a cohort study which aimed to characterize the pharmacokinetics of recombinant human factor VIIa (rFVIIa) in haemophilia patients, 10 subjects with severe FVIII or FIX deficiency were treated with rFVIIa [55]. The rFVIIa–AT complex formation was responsible for 65% of the total rFVIIa clearance. The formation of the FVII–AT complex was able to explain the difference observed between the recombinant FVIIa activity (rFVIIa:C) and the antigen (rFVII:Ag) concentration [55]. In a recent study on venous thrombotic risk in patients with antineutrophil cytoplasmic antibody (ANCA)-associated vasculitis, coagulation parameters were assessed at presentation with active disease and at follow-up after 6 months of treatment in 71 patients [56]. FVIIa–AT levels were normal in the majority of patients (90%) at baseline and further slightly decreased over time. Four of 71 (6%) had a history of VTE and five had VTE symptomatic after presentation. No difference in FVIIa–AT levels was found between patients with or without VTE [56]. Finally, within the frame of the previously mentioned Cardiovascular Health Study (CHS), in a cohort of adults aged 65 years and older and free of dementia in 1992/1993 (n = 3185), there was no evidence of linear associations of FVIIa–AT levels with any dementia risk [57].

## 5. Correlations with Other Clinical and Laboratory Parameters

FVIIa–AT levels have been found to correlate directly with peak and endogenous thrombin potential (ETP) levels by thrombin generation analysis, with high FVIIa–AT plasma concentration associated with increased peak and ETP levels [31]. Similarly, high FVIIa–AT plasma levels were related to increased FXa generation, with hypercoagulability features specifically detectable in the coagulation initiation phase [32]. Such results consistently indicate FVIIa–AT as a thrombophilic biomarker, with high levels correlated with a procoagulant/prothrombotic diathesis. Beyond the expected and strong correlation with FVII/FVIIa, which was often the main predictor of FVIIa–AT variability [31,33], FVIIa–AT plasma levels have been found to relate to other clinical and laboratory parameters. An inverse correlation with renal function (the lowest the renal function, the highest the FVIIa–AT levels) has been described [31]. Similarly, chronic kidney disease is well known to be associated with an increase in FVII activity due not only to altered renal clearance but also to potentially procoagulant endothelial dysfunction [58]. Multiple direct correlations of FVIIa–AT with plasma lipid and apolipoprotein profile (total and high-density lipoprotein cholesterol, triglycerides, ApoA-I, ApoC-III, and ApoE) have been reported [31,33,44] and, notably, low-density lipoprotein (LDL) receptor-related protein 1 (LRP1) may contribute to the clearance of FVIIa–AT complex in vivo [59]. Direct correlation with inflammatory markers, like C reactive protein (CRP), has been also reported, as well as direct associations with current smoking, systolic blood pressure, and female gender [33]. On the one hand, all these associations support the concept that different conditions, including many traditional cardiovascular risk factors, are associated with an overall increased TF expression/activity. On the other hand, such associations should remind us how FVIIa–AT plasma levels can be influenced by multiple conditions that can act as possible confounding factors in evaluating its real clinical significance and prognostic impact. However, it is worth noting that, in the prospective and longitudinal studies evaluating the prognosis in patients with cardiovascular disease or cancer, high FVIIa–AT plasma levels remained a significant and independent predictor of mortality, even after multiple adjustments for potential concomitant risk factors [31,33,41].

## 6. Strengths, Limitations, and Perspectives for Using FVIIa–AT to Explore the TF-Related Pathway

FVIIa–AT plasma concentrations, as an indirect marker of TF–FVIIa interaction, may overcome the difficulties related to the evaluation of a transmembrane molecule like TF, which is usually not directly detected in plasma (with the notable exception of EV-associated TF), has alternatively spliced forms (like the soluble asTF), and exists in different (encrypted and decrypted) conformations. As regards the last issue, it should be noted that TF conformations are not discriminated by antigenic assays which cannot distinguish the different isoforms and measure all forms of TF without giving functional information.

However, there are certainly many significant limitations which should be taken into account when considering FVIIa–AT as a potential biomarker of the TF pathway:

(i) FVIIa–AT plasma levels can be informative on the global TF–FVIIa interaction but are unable to disentangle the different cellular origins of TF expression/activity (e.g., EVs, platelets, monocytes, activated endothelial cells, neoplastic cells, etc.) which may have significant pathophysiological and different clinical implications;
(ii) The method for measuring FVIIa–AT complex concentration requires standardisation, for the pre-analytical and analytical phases, in order to ensure greater reproducibility and comparability of the results. Taking into account that activated platelets can express TF [60] and how platelets can be triggered/activated during blood drawn [61], the compliance with procedures to limit platelet activation during blood sampling and the appropriate preparation of platelet-poor plasma (PPP) samples would be crucial to avoid further confounding elements in FVIIa–AT evaluation;
(iii) Up to now, all the studies in humans showed a large range of overall distribution of FVIIa–AT plasma levels, as well as a substantial overlap of values between pathological cases and healthy controls. Therefore, any potential for diagnostic sensitivity and specificity appears to be limited;
(iv) The plasma levels of FVIIa–AT are strongly influenced by those of FVII/FVIIa, which have been indicated among the main determinants of FVIIa–AT concentration in many studies [25,31,33]. Consistently, several clinical conditions are recognized to be related to low levels of FVII/FVIIa by means of either reduced synthesis (e.g., genetic variants leading to low FVII expression, liver cirrhosis, VKAs therapy) or increased consumption (e.g., acute thrombosis, disseminated intravascular coagulation), and have been also associated with low levels of FVIIa–AT [25,28,29,30,33]. Such decrease, which is mediated predominantly by FVII levels (and not by TF–FVIIa interaction), could affect the value of the FVIIa–AT assay as an efficacious biomarker of TF activity and likely limits its prognostic role. For instance, in previous studies, FVIIa–AT discriminated between subjects with or without portal vein thrombosis only among non-cirrhotic subjects, while cirrhotic patients with or without portal vein thrombosis had similarly very low levels of FVIIa–AT [28,29];
(v) Many traditional laboratory parameters, from renal function to plasma lipid profile, have been shown to predict FVIIa–AT variability, also consistently with the effects on FVII/FVIIa, thus raising the clinical suspicion that the FVIIa–AT-related prognostic role may be merely an epiphenomenon of the changes and imbalances of other traditional and crucial cardiovascular risk factors [31,43,44].

According to all these considerations, it could be hypothesized that, in order to optimise the relevance of FVIIa–AT as an indirect marker of TF activity, the sampling time for plasma assays should be after a night of fasting (to avoid the potential interference of fat-containing meals) in clinically stable subjects (without any acute thrombotic events), not taking drugs reducing FVII levels (like VKAs) or with concomitant medical condition reducing FVII and FVIIa–AT levels (like advanced liver disease or disseminated intravascular coagulation). The potential influence related to renal failure and hyperlipidaemia, both associated with higher FVIIa–AT levels, should also be taken into account.

Nonetheless, in spite of all these limitations and confounding factors, it is intriguing to observe that the levels of FVIIa–AT are increased in many different illnesses, from cardiovascular to thrombotic diseases, from cancer to infection, and—most importantly—such an increase could predict a worse prognosis in the specific clinical settings [31,33,41]. Actually, it appears biologically plausible that TF overexpression could be unfavourable and potentially detrimental in pathophysiological pathways characterized by prothrombotic diathesis, excessive stimulation of inflammatory pathways, or inappropriate cellular proliferation. From a pragmatic point of view, the capability of the FVIIa–AT complex to be a global marker of the TF–FVIIa interaction, even if not able to better characterize and differentiate the cellular origins of such an interaction, could allow us to intercept a background signal of hyperactivation of the TF pathway and potentially overcome the limitations related to a restricted assessment of TF activity (e.g., that related to extracellular vesicles (EVs)). In our previous work in patients with liver cancer, FVIIa–AT levels but not EV-associated TF-dependent procoagulant activity predicted the risk of overall mortality and no correlation was found between FVIIa–AT levels and EV-associated TF-dependent procoagulant activity, thus suggesting that, in this study population, TF-positive EVs were not the main source of TF–FVIIa interaction [41].

**Figure 4:**
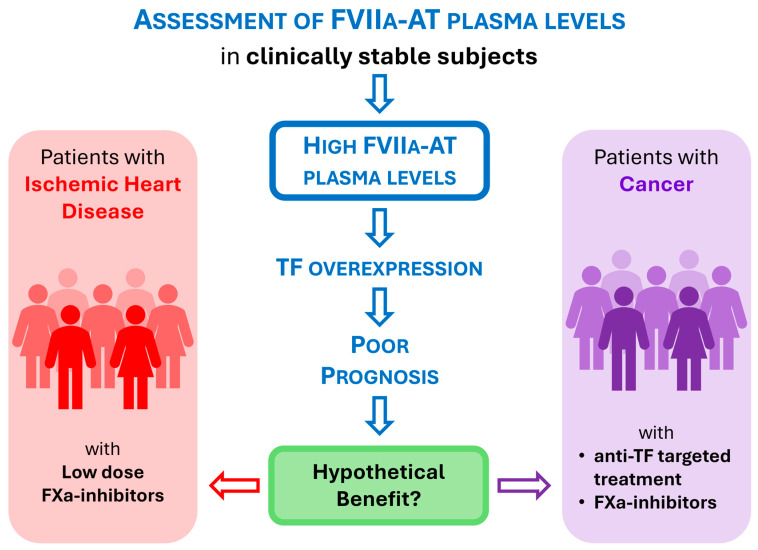
Potential benefit of plasmatic FVIIa–AT assessment in clinical practice. Hypothesis of the use of plasma FVIIa–AT levels in the management of patients with different medical conditions (cardiovascular disease/cancer). Speculatively, cardiovascular patients with high FVIIa-AT levels could have a more favourable benefit/risk ratio from treatment with low dose of FXa-inhibitors [62]. Similarly, cancer patients with high FVIIa-AT levels could have a more favourable benefit/risk ratio from treatment with FXa-inhibitors [65] or with specific anti-TF targeted therapies [66].

Notably, a laboratory biomarker able to identify patients with high expression/TF activity may have not only prognostic but also therapeutic implications and may pave the way for more patient-tailored management of disease. In 2016, according to the prognostic role of FVIIa–AT in our cohort of patients with clinically stable CAD, we hypothesized the possible beneficial use of oral direct FXa inhibitors in patients with high FVIIa–AT levels, by blocking the excess of FX activation mediated by the FVIIa–TF pathway [31]. In 2017, the COMPASS trial showed that low doses of the FXa-inhibitor rivaroxaban, 2.5 mg twice daily, plus aspirin, led to better cardiovascular outcomes than aspirin alone in patients with stable atherosclerotic vascular disease; although, it also increased the risk of major bleeding [62]. Now, we could further speculate that FVIIa–AT assessment may allow the identification of the subgroup of stable CAD patients who can have the major benefit from a more aggressive antithrombotic treatment associating antiplatelet agents and FXa-inhibitors (Figure 4).

FXa-inhibitors have been also proposed to have an anti-neoplastic role, consistent with the observation that coagulation factors contribute to cancer immune evasion [63,64]. The FXa-inhibitor rivaroxaban has been shown to have anti-neoplastic synergistic effects with immune checkpoint inhibitors (ICIs) in a real-world cohort of patients with metastatic melanoma [65]. According to these considerations, also in the setting of neoplastic disease, we could hypothesize that high FVIIa–AT plasma levels may allow us to identify patients with cancer overexpressing TF who can receive major benefits from such treatment approaches or specifically from anti-TF molecularly targeted treatment against malignant cells (Figure 4). With regards to the latter issue, it is worth noting that tisotumab vedotin, an antibody–drug conjugate combining an anti-TF monoclonal antibody and an inhibitor of cell division, has been approved for the treatment of cervical cancer as a first-in-class medication [66,67].

## 7. Conclusions

In summary, FVIIa–AT plasma concentrations appear so far as an imperfect though biologically sound biomarker of a TF-related pathway that can be clinically useful given that elevated levels are associated with procoagulant diathesis and worse clinical outcomes. Current data so far do not suggest diagnostic relevance, consistently with the lack of specificity and the wide distribution range of this biomarker, but rather a potential role in the prognostic stratification of patients with well-defined clinical conditions in whom TF overexpression may have harmful effects (like cardiovascular disease or cancer). Certainly, the biological and clinical role of FVIIa–AT should be further investigated and eventually validated by future larger studies, preferentially with prospective design and taking into account the large variability and the multiple conditions potentially influencing this biomarker. Nonetheless, the present data suggest that the assessment of FVIIa–AT, when properly weighed according to the clinical settings and avoiding potential confounding factors, may contribute to shedding light on the relevance of the TF-related pathway in health and disease and, thereby, better and more patient-tailored disease management. This could be obtained, speculatively, by identifying subjects with high TF expression/activity who may have the most favourable benefit–risk ratio when considering treatment with—for instance—either FXa inhibitors or TF-directed therapies (Figure 4).

## Data Availability

No new data were created or analyzed in this study. Data sharing is not applicable to this article.
